# Supplementary material for: Brain-infiltrating CD4 T cells drive inflammatory microglia proliferation during cryptococcal meningitis in mice
Source: Nat Commun. 2025 Oct 9;16:8995. doi: 10.1038/s41467-025-64034-5 (PMC12511619; doi:10.1038/s41467-025-64034-5)
Supplement: Supplementary file 1 — Supplementary Information [file 41467_2025_64034_MOESM1_ESM.pdf]

Supplemental Figures and Legends

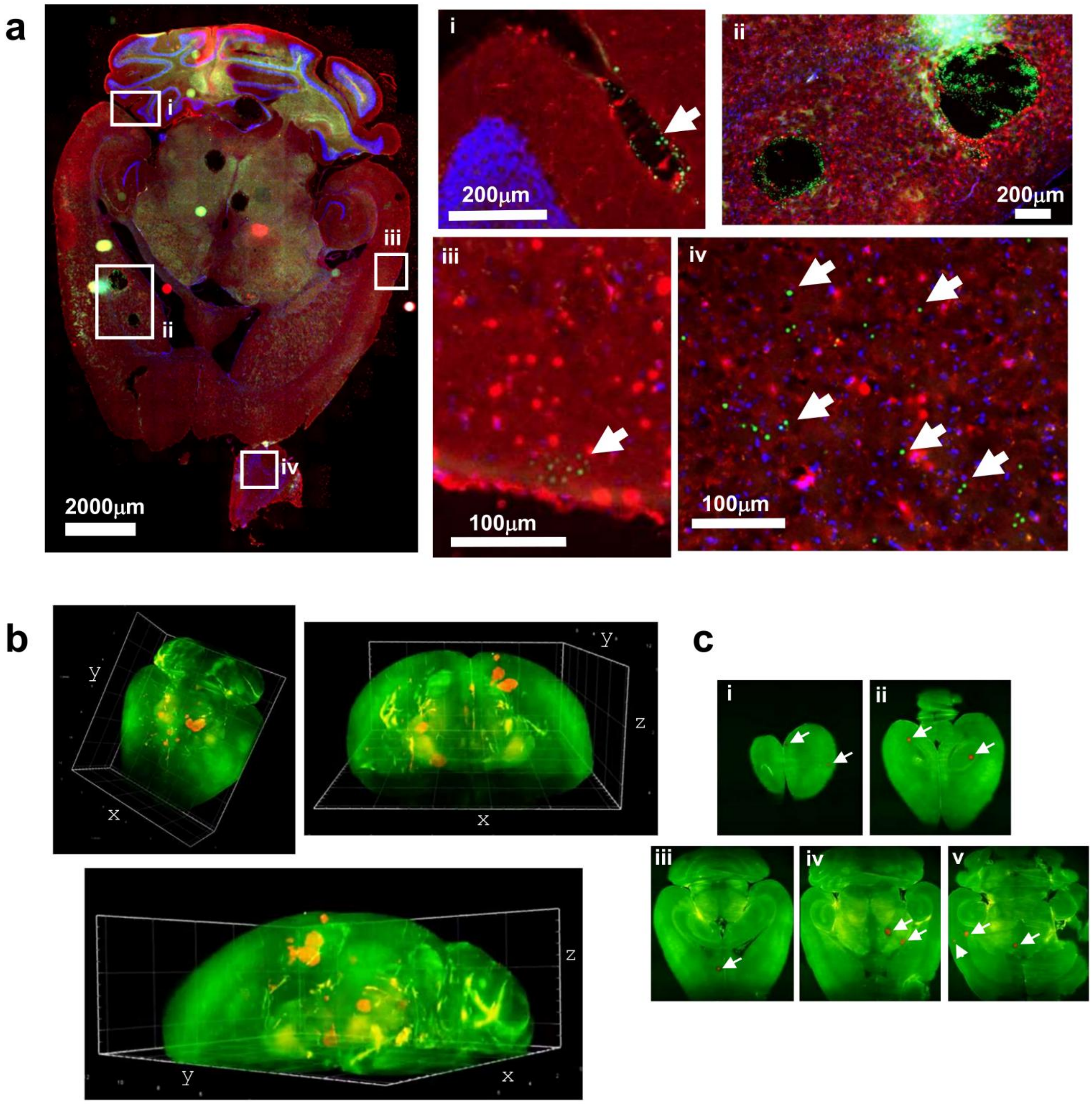

Fig S1: Example 2D (confocal) and 3D (lightsheet) microscopy from day 7 post-infection highlighting areas of fungal infection and their location within the brain. (a) Confocal microscopy of *C. neoformans* infected brain from day 7 post intravenous infection with GFP-expressing *C. neoformans*, stained with anti-Iba1 (red, microglia marker), DAPI nuclear stain (blue). Insets show enlarged areas of infection within the (i) cerebellum, (ii) caudate putamen, (iii) cerebral cortex, and (iv) nasal bulb. (b) Light-sheet imaging of mouse brain at day-7 post-infection with mCherry-expressing *C. neoformans* (red areas). Background autofluorescence of the brain tissue shown in green. (c) Sequential sections (i-v) through brain tissue imaged by light-sheet microscopy, with fungal lesions highlighted with white arrows. Example images are representative of at least two brains from individual mice analysed.

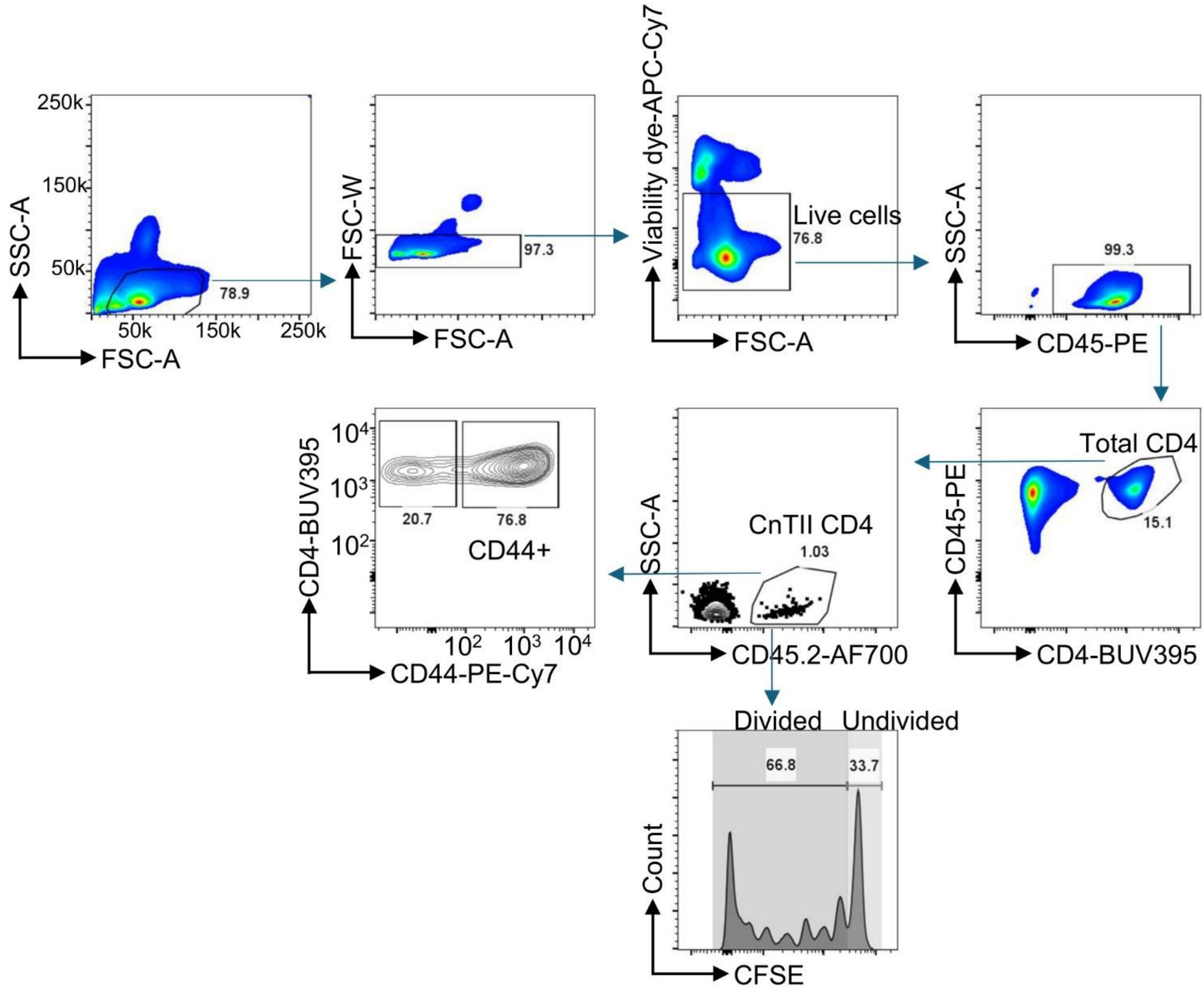

Fig S2: Gating strategy for total CD4 T cells and adoptively transferred CnT.II T cells, including example plots of staining for activation marker CD44 and proliferation dye CFSE.

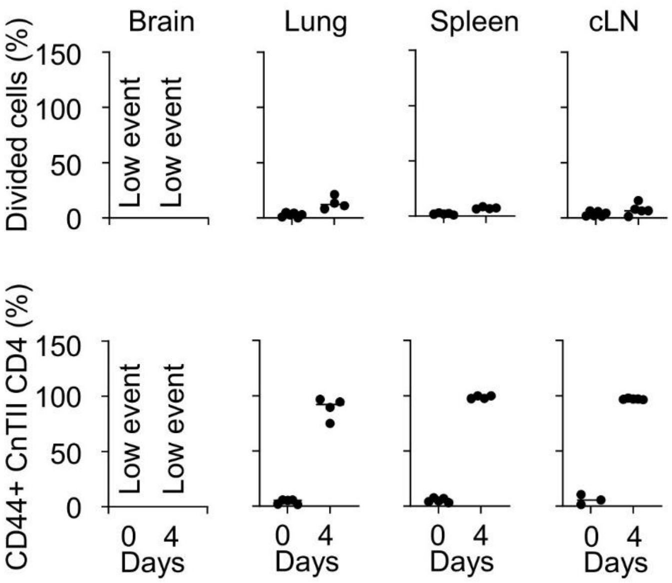

Fig S3: Division (CFSElow cells) and CD44 expression of transferred CnT.II cells in uninfected (n=6 mice) and infected (day 4 post-infection; n=7 mice) mice. In mice where there were <50 CnT.II cells within the gate, these mice were excluded from analysis. This removed all brain samples. Data pooled from two independent experiments.

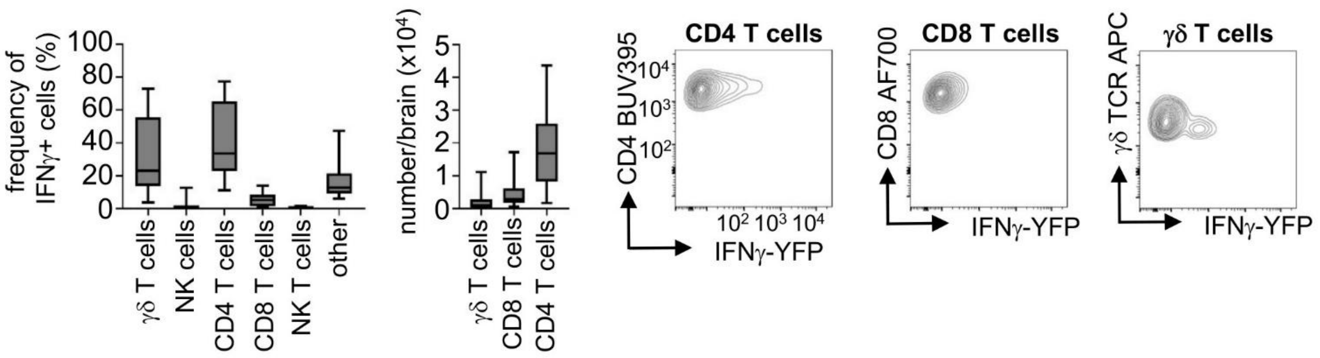

Fig S4: IFNγ-YFP reporter animals (n=14 mice) were infected intravenously with *C. neoformans* and brain cells analysed by flow cytometry at day 7 post-infection. Total IFNγ-YFP+ cells were first gated and indicated cell populations identified using lineage marker staining (γδ T-cells, γδ TCR+CD3+; CD4 T cells, CD4+CD3+; CD8 T cells, CD8+CD3+, NK cells, NK1.1+CD3-; NKT cells, NK1.1+CD3+). Alternatively, cell types were first gated and these frequencies used to calculate total numbers of indicated cell types. Example plots are gated on indicated cell types to show IFNγ-YFP signal within each population. Data pooled from 2 independent experiments. In all box-and-whisker plots, whiskers refer to the maximum/minimum values, the box refers to interquartile ranges, the centre line refers to the mean.

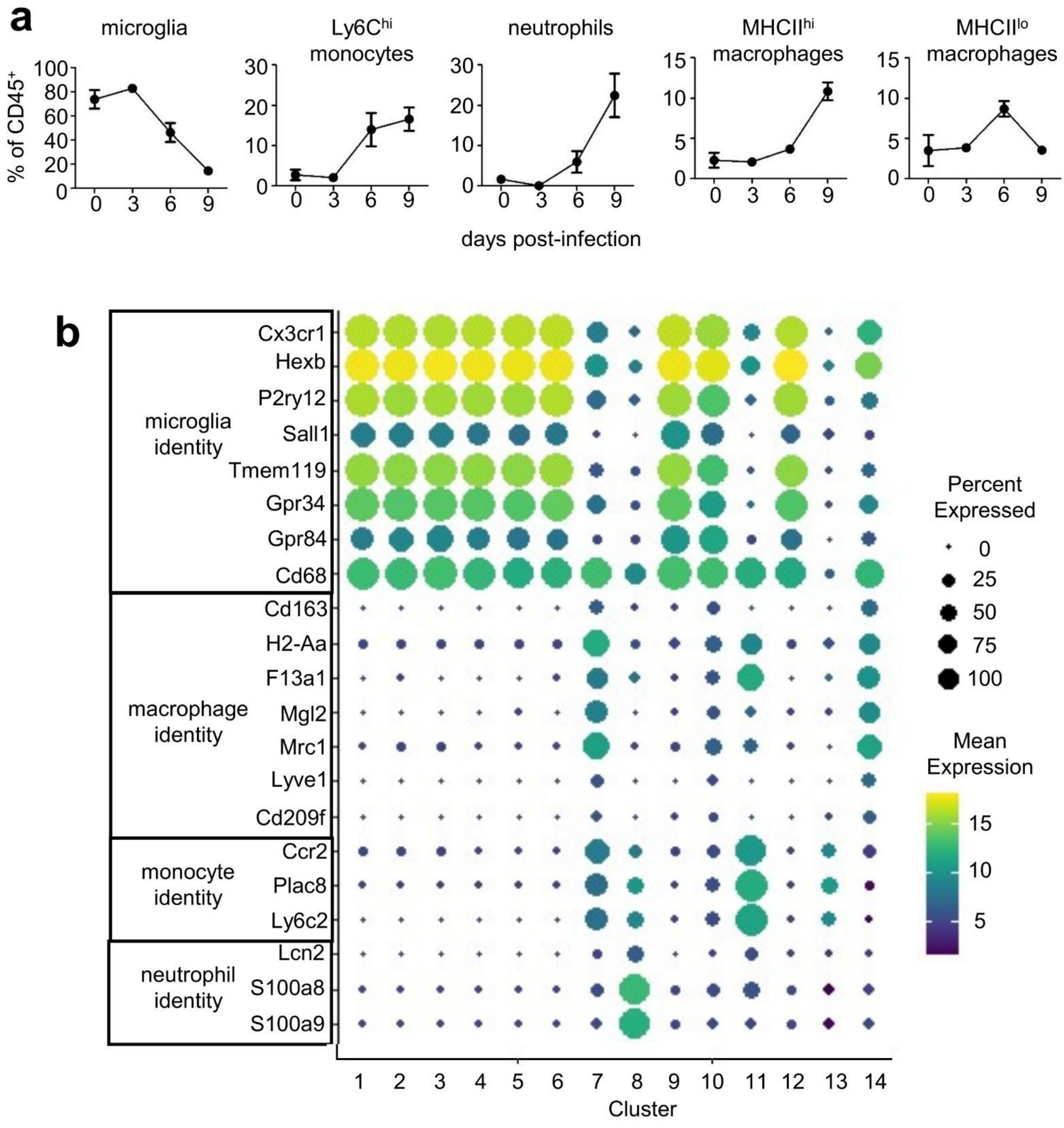

Fig S5: (a) Proportions of major myeloid cell subsets (as determined by flow cytometry) in the brain at days 0, 3, 6 (n=3 mice per time point) and 9 (n=4 mice) post-infection. Data from a single experiment, n = 3-4 mice per time point. Data presented as mean +/- SEM. (B) Bubble plot showing percent and mean expression level of indicated genes within the cell clusters identified by graph-based clustering on the pooled dataset.

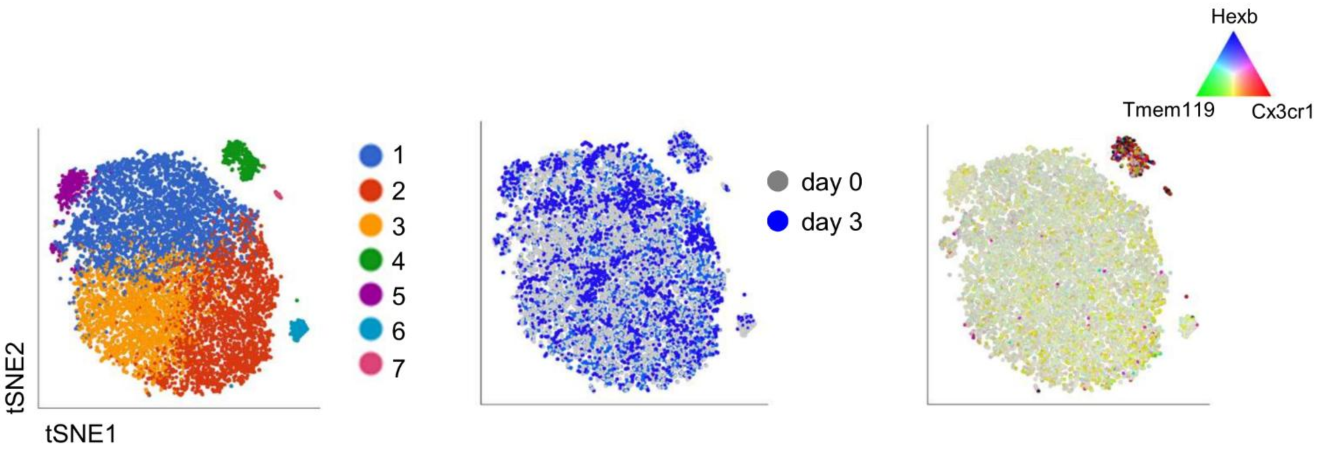

Cluster 1	Cluster 2	Cluster 3	Cluster 4	Cluster 5	Cluster 6	Cluster 7
Hsd17b4	Gm26520	Fos	H2-Aa	Ppp1r14b	Malat1	Chil3
Gm13339	Gm3033	Atf3	H2-Eb1	Erdr1	Csf1r	Ly6c2
4930589L23Rik	Gm24447	Dusp1	Pf4	Csf1	Serinc3	Plac8
Poc1a	1700028E10Rik	Jun	Mgl2	Cmip	Ctsd	S100a4
Tmsb15l	Gm24265	Zfp36	Ms4a7	Marcksl1	Lgmn	Hp
Cox20	Ttc14	Junb	Cd74	Hnrnpa0	C1qa	S100a6
Ttn	Zfp273	Egr1	H2-Ab1	Mapkapk2	Ctsb	Napsa
Gm3534	RP24-142B15.4	Jund	Cxcl2	Reep4	Gm26532	Fn1
Gm8181	RP23-326O2.2	Klf2	Cd209f	Rn18s-rs5	Fcrls	Mgst1
Gm11769	BC068281	Ubc	Cbr2	Tnf	Selplg	Lgals3

Fig S6: Analysis of uninfected and day 3 post-infection samples, tSNE plots coloured by graph-based clustering results, sample distribution, and microglia signature gene expression. The table below shows the top 10 biomarkers for each of the 7 clusters identified by graph-based clustering, identifying cluster 4 as macrophages and cluster 7 as monocytes – all others are microglia.

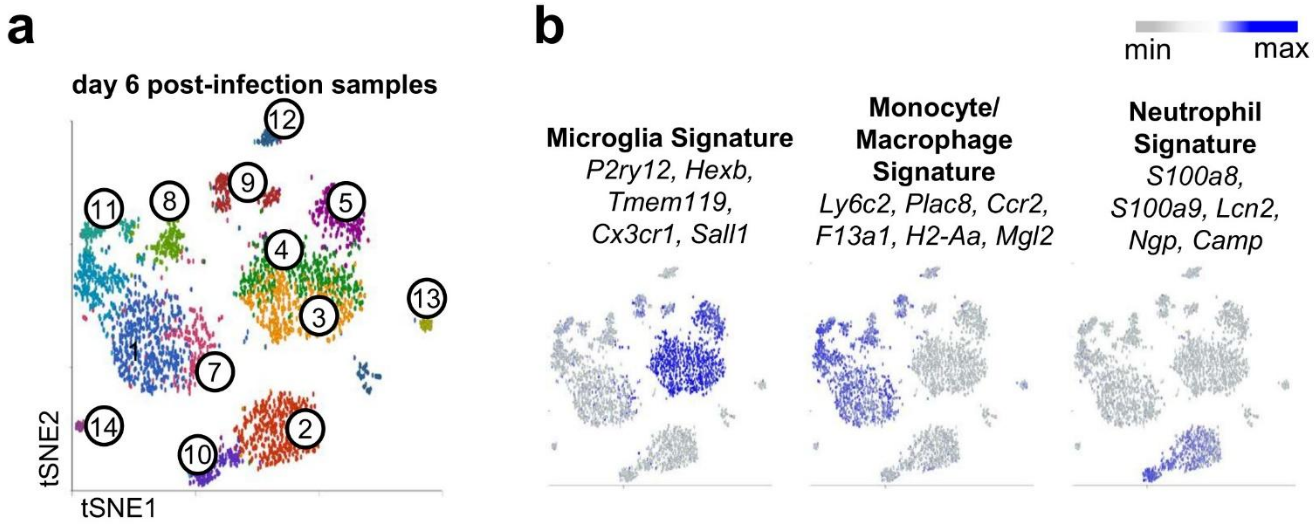

c

**Microglia Clusters**		
3	4	5
Tmem119	Tmem119	Gpr84
Cx3cr1	Gpr34	Ccl12
P2ry12	Siglech	Nop56
Sparc	P2ry12	Ppan
Hexb	Sparc	Srm
Gpr34	Olfml3	Il1a
Olfml3	Cx3cr1	Ccdc86
C1qa	Hexb	Erdr1
Cd81	Ltc4s	Wdr12
C1qc	Cd81	Impdh2

**Neutrophil Clusters**	
2	10
Ccrl2	Lrg1
Irg1	Retnlg
Gadd45b	Pglyrp1
Cd24a	S100a9
S100a8	Hdc
S100a9	S100a8
Il1f9	Mmp9
Csf1	Wfdc21
Il1rn	Lcn2
Hcar2	G0s2

**Monocyte/Macrophage Clusters**					
1	6	7	8	9	11
Arg1	Ifi203	Arg1	Ccl17	Pf4	Clec4a1
Ccl24	Mndal	Mrc1	Il4i1	Cbr2	Rasgrp2
Fn1	Fam26f	Ccl24	Ccl22	Lyve1	Napsa
F10	Plac8	Fn1	Ramp3	Ms4a7	Stap1
Dab2	Pyhin1	Fabp5	H2-DMb2	Folr2	Itgb7
AA467197	Ms4a4c	F7	Kmo	Cp	Fam69a
Tgfbi	Ifi205	Spp1	Traf1	Apoe	Cybb
Sdc4	Vcan	Myof	H2-Eb1	Fcna	Cbfa2t3
Slc7a2	Cybb	Rnase2a	Ccnd2	Igfbp4	S100a4
Tgm2	Slfn5	Vat1	H2-Aa	Igf1	Itga4

Fig S7: Analysis of day 6 post-infection samples showing (a) graph-based clustering result, (b) signature gene expression, and (c) the top 10 biomarkers for indicated clusters.

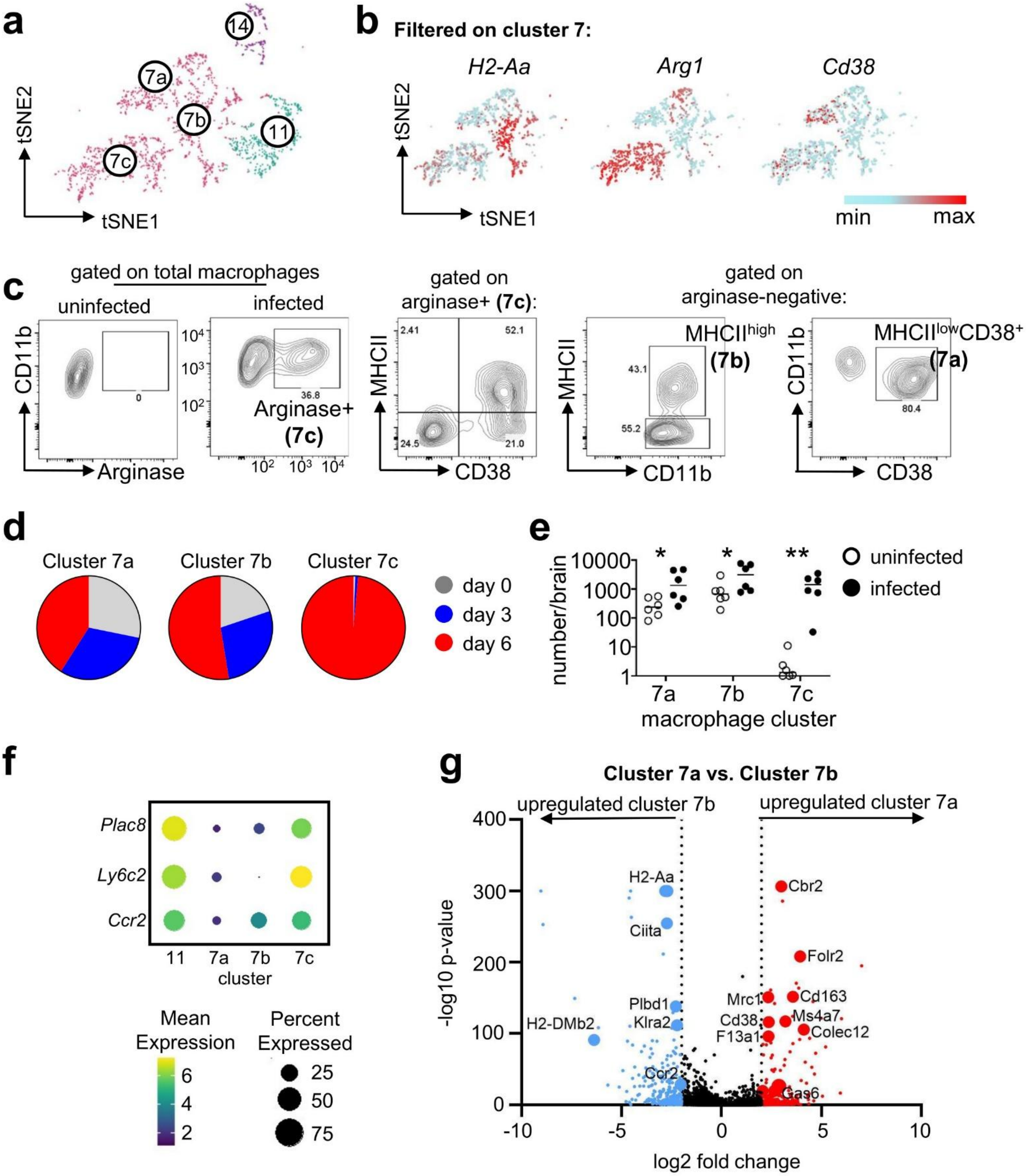

Fig S8: (a) tSNE plot of macrophage and monocyte clusters (7, 11 and 14). (b) Expression of *H2-Aa, Cd38* and *Arg1* by macrophage cluster 7. (c) Example flow cytometry plots showing gating strategy for the three major subsets of identified macrophages within cluster 7. (d) Proportions of sequenced cells from analysed time points post-infection for each of the cluster 7 sub-clusters. (e) Numbers of each macrophage sub-cluster as determined by flow cytometry using the gating strategy shown in (c). Data pooled from 2 independent experiments, each dot represents an individual mouse (n=6 uninfected mice [open symbols], n=6 infected mice [closed symbols]) and line represents the mean. Data analysed by Mann Whitney U-tests, **P*=0.0411, ***P*=0.0022. (f) Mean expression and percent of cells with transcripts for the indicated monocyte signature genes in each of the macrophage sub-clusters, with cluster 11 (monocytes) shown as a positive control. (g) Volcano plot showing differentially-expressed genes between macrophage clusters 7a and 7b. Genes that were

previously identified as markers for MHCII^{hi} and MHCII^{lo} border macrophages[1] are highlighted with larger dots and labelled.

[1]Van Hove et al (2019) Nature Neuroscience, 22: 1021-1035. (this published dataset was used for comparison and identification of border macrophage subpopulations in the mouse brain)

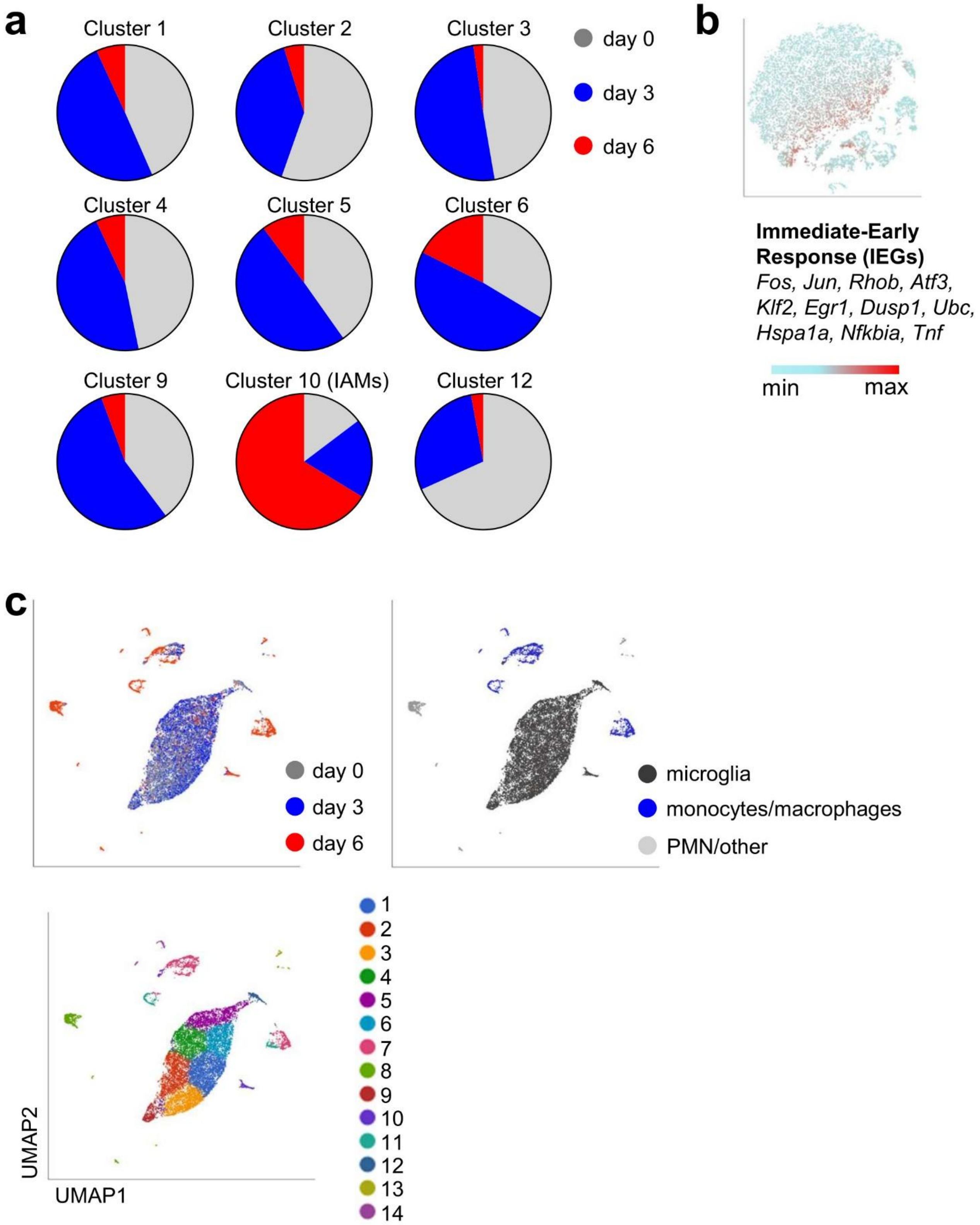

Fig S9: (A) Pie charts showing proportion of each identified microglia cluster (see Fig S5) from uninfected, day 3 post-infection and day 6 post-infection samples. (B) tSNE plot coloured by mean expression of indicated immediate-early response genes. (C) UMAP visualisation of dataset, coloured by time point post-infection, cell identity and graph-based clustering result.

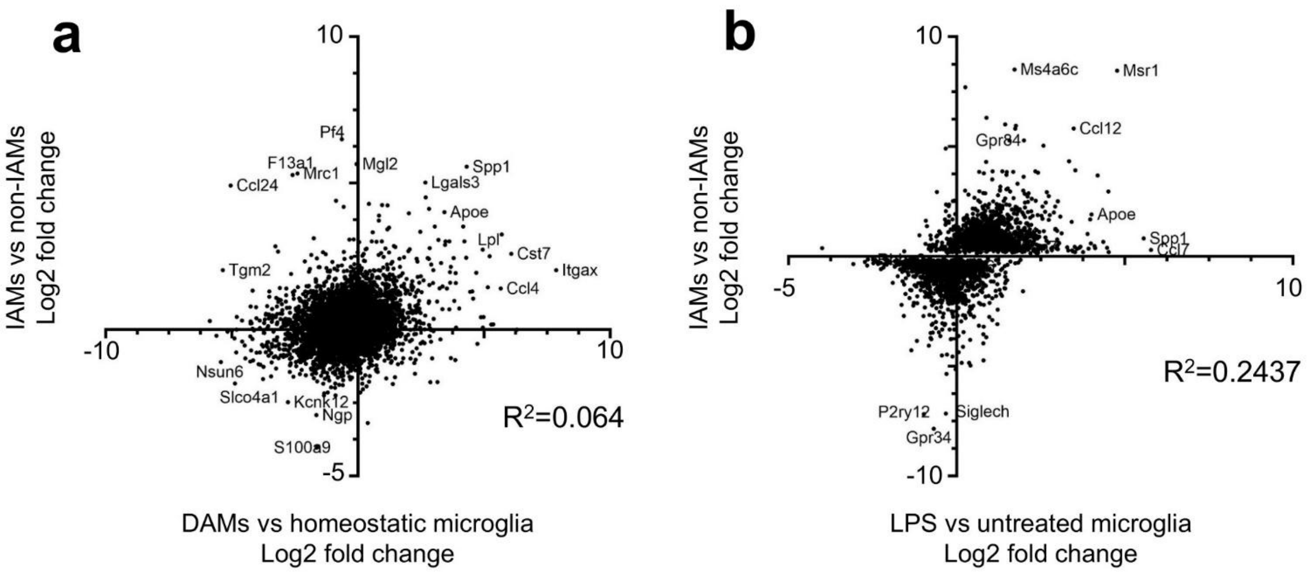

Fig S10: Comparison of upregulated and downregulated genes between IAMs and non-IAMs (our dataset) with gene expression patterns in damage-associated microglia (DAMs; from dataset published in Keren-Shaul et al 2017) or LPS-associated microglia (from dataset published in Sousa et al 2018). Data analysed by simple linear regression.

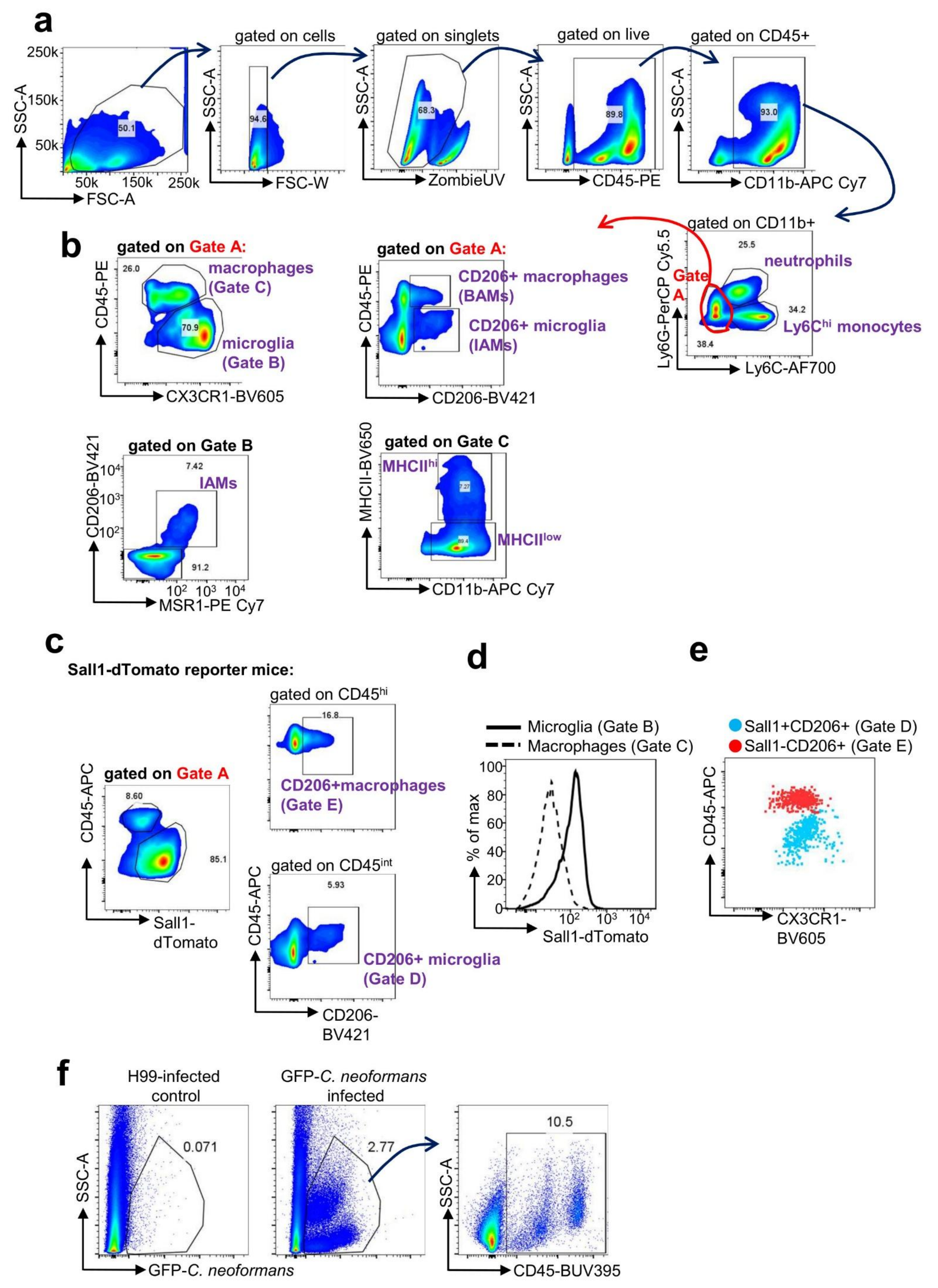

Fig S11: Myeloid gating strategy. (a) Basic gating strategy to identify live CD45$^+$ singlets, then CD11b$^+$ myeloid cells which differentiated three main populations, Ly6G$^+$ neutrophils, Ly6C^{hi} monocytes and 'other' (Gate A). (b) Within Gate A, microglia and border macrophages are

differentiated by CD45 and CX3CR1 expression. MHCIIhi and MHCIIlo macrophages are found within the $CD45^{hi}$ gate (Gate C), as are $CD206^{hi}$ border macrophages. IAMs co-express CD206 and MSR1 and are found within the $CD45^{int}$ gate (Gate B). (c) Example gating and plots using Sall1-dTomato reporter mice. (d) Sall1 expression is limited to the $CD45^{int}$ gate (Gate B) and not found within macrophages ($CD45^{hi}$, Gate C). (e) Back gating of $CD206^{+}CD45^{int}$ microglia (Gate D) and $CD206^{+}CD45^{hi}$ macrophages (Gate E) to demonstrate that these are two distinct populations that can be separated based on differential CD45 expression. (f) Example plots showing gating for GFP-expressing *C. neoformans* in the brain, compared to control animal that was infected with wild-type *C. neoformans*. CD45+ populations within the fungal-positive population was denoted as the 'intracellular' or host-cell associated yeast population.

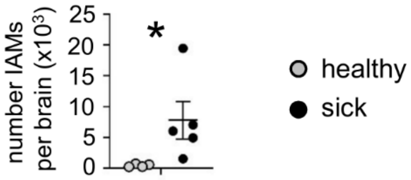

Fig S12: Quantification of IAMs (CD206^{+}MSR1^{+}CD45^{int}CX3CR1^{high} microglia) in the brains of mice infected with *Blastomyces dermatitidis*. Animals were euthanised upon developing neurological symptoms and signs of illness (‘sick’ group, closed symbols) on day 4 or 6 post-infection. Each point represents an individual animal (n=4 healthy mice [open symbols], n=5 sick mice). Data analysed by Mann Whitney U-test. *P=0.0159.
