# Supplementary material for: Brain-infiltrating CD4 T cells drive inflammatory microglia proliferation during cryptococcal meningitis in mice
Source: Nat Commun. 2025 Oct 9;16:8995. doi: 10.1038/s41467-025-64034-5 (PMC12511619; doi:10.1038/s41467-025-64034-5)
Supplement: Supplementary file 2 — Description of Additional Supplementary Files [file 41467_2025_64034_MOESM2_ESM.pdf]

Description of Additional Supplementary Files

Supplementary Data 1: List of differentially-expressed genes between IAMs (cluster 10) and non-IAM microglia clusters. Genes are listed with the mean expression value for each population, the fold change and *P*-value. Statistical analysis was performed using default settings within the DESeq2 pipeline in Partek Flow, which uses the Wald test with FDR multiple correction.

Supplementary Data 2: List of cell-cycle genes present in the sequencing dataset, their classification for cell-cycle stage and whether reads for indicated gene were detected in IAMs population (cluster 10).
