# Supplementary material for: Brain-infiltrating CD4 T cells drive inflammatory microglia proliferation during cryptococcal meningitis in mice
Source: Nat Commun. 2025 Oct 9;16:8995. doi: 10.1038/s41467-025-64034-5 (PMC12511619; doi:10.1038/s41467-025-64034-5)
Supplement: Supplementary file 5 — Reporting Summary [file 41467_2025_64034_MOESM5_ESM.pdf]

nature portfolio

Corresponding author(s): Rebecca Drummond

Last updated by author(s): 2025/08/27

# Reporting Summary

Nature Portfolio wishes to improve the reproducibility of the work that we publish. This form provides structure for consistency and transparency in reporting. For further information on Nature Portfolio policies, see our Editorial Policies and the Editorial Policy Checklist.

## Statistics

For all statistical analyses, confirm that the following items are present in the figure legend, table legend, main text, or Methods section.

| n/a | Confirmed | |
|---|---|---|
| ☐ | ☒ | The exact sample size (*n*) for each experimental group/condition, given as a discrete number and unit of measurement |
| ☐ | ☒ | A statement on whether measurements were taken from distinct samples or whether the same sample was measured repeatedly |
| ☐ | ☒ | The statistical test(s) used AND whether they are one- or two-sided<br>*Only common tests should be described solely by name; describe more complex techniques in the Methods section.* |
| ☒ | ☐ | A description of all covariates tested |
| ☐ | ☒ | A description of any assumptions or corrections, such as tests of normality and adjustment for multiple comparisons |
| ☐ | ☒ | A full description of the statistical parameters including central tendency (e.g. means) or other basic estimates (e.g. regression coefficient) AND variation (e.g. standard deviation) or associated estimates of uncertainty (e.g. confidence intervals) |
| ☐ | ☒ | For null hypothesis testing, the test statistic (e.g. *F*, *t*, *r*) with confidence intervals, effect sizes, degrees of freedom and *P* value noted<br>*Give P values as exact values whenever suitable.* |
| ☒ | ☐ | For Bayesian analysis, information on the choice of priors and Markov chain Monte Carlo settings |
| ☒ | ☐ | For hierarchical and complex designs, identification of the appropriate level for tests and full reporting of outcomes |
| ☒ | ☐ | Estimates of effect sizes (e.g. Cohen's *d*, Pearson's *r*), indicating how they were calculated |

*Our web collection on statistics for biologists contains articles on many of the points above.*

## Software and code

Policy information about availability of computer code

| | |
|---|---|
| Data collection | BD FACS Diva v9, Zen Black v3.1, INSPIRE ImageStream Data Acquisition Software, CellRanger v3.0 |
| Data analysis | FlowJo v10, GraphPad Prism v10, Image J, Zen Blue v3.1, IDEAS v6.2, Partek Flow v9.0.20 |

For manuscripts utilizing custom algorithms or software that are central to the research but not yet described in published literature, software must be made available to editors and reviewers. We strongly encourage code deposition in a community repository (e.g. GitHub). See the Nature Portfolio guidelines for submitting code & software for further information.

## Data

Policy information about availability of data

All manuscripts must include a data availability statement. This statement should provide the following information, where applicable:

- Accession codes, unique identifiers, or web links for publicly available datasets
- A description of any restrictions on data availability
- For clinical datasets or third party data, please ensure that the statement adheres to our policy

All raw data associated with the figures are without restriction. Source data are provided with this paper. Raw sequencing data and processed data files have been deposited in the GEO database under accession number GSE262502 [https://www.ncbi.nlm.nih.gov/geo/query/acc.cgi?acc=GSE262502].

## Research involving human participants, their data, or biological material

Policy information about studies with human participants or human data. See also policy information about sex, gender (identity/presentation), and sexual orientation and race, ethnicity and racism.

| | |
|---|---|
| Reporting on sex and gender | N/A |
| Reporting on race, ethnicity, or other socially relevant groupings | N/A |
| Population characteristics | N/A |
| Recruitment | N/A |
| Ethics oversight | N/A |

Note that full information on the approval of the study protocol must also be provided in the manuscript.

# Field-specific reporting

Please select the one below that is the best fit for your research. If you are not sure, read the appropriate sections before making your selection.

- [x] Life sciences
- [ ] Behavioural & social sciences
- [ ] Ecological, evolutionary & environmental sciences

For a reference copy of the document with all sections, see nature.com/documents/nr-reporting-summary-flat.pdf

# Life sciences study design

All studies must disclose on these points even when the disclosure is negative.

| | |
|---|---|
| Sample size | In each of the animal experiments, we aimed to have n=6 per experiment group, since our power calculations indicated that this sample size would allow for a detection of a 30% difference in the means with a probability of greater than 95%, assuming a standard deviation of around 19% and minimum power 0.8. These calculations are based on years of experience with these models and measuring these parameters using our chosen methods. |
| Data exclusions | No data exclusions. |
| Replication | All experiments were repeated at least twice and up to 4 times, to ensure reproducibility of the results. Data from each experiment was pooled and raw data is presented in the figures and in the Source Data File. |
| Randomization | In all experiments, the assignment of littermate controls to cages, the placement of the experimental cage within the animal facility and the order in which infections/analysis procedures were performed were done at random. |
| Blinding | Blinding was not appropriate for the current study design and in many experiments, not possible due to nature of mouse breeding. |

# Reporting for specific materials, systems and methods

We require information from authors about some types of materials, experimental systems and methods used in many studies. Here, indicate whether each material, system or method listed is relevant to your study. If you are not sure if a list item applies to your research, read the appropriate section before selecting a response.

## Materials & experimental systems

| n/a | Involved in the study | |
|---|---|---|
| [ ] | [x] | Antibodies |
| [x] | [ ] | Eukaryotic cell lines |
| [x] | [ ] | Palaeontology and archaeology |
| [ ] | [x] | Animals and other organisms |
| [x] | [ ] | Clinical data |
| [x] | [ ] | Dual use research of concern |
| [x] | [ ] | Plants |

## Methods

| n/a | Involved in the study | |
|---|---|---|
| [x] | [ ] | ChIP-seq |
| [ ] | [x] | Flow cytometry |
| [x] | [ ] | MRI-based neuroimaging |

## Antibodies

| | |
|---|---|
| Antibodies used | CD45 (30-F11), CD11b (M1/70), CX3CR1 (SA011F11), MHC Class II (M5/114.15.2), F480 (BM8), Ly6G (1A8), Ly6C (HK1.4), CD44 (IM7), |

CD69 (H1.2F3), CD127 (A7R34), CD45.2 (A20), CD45.1 (104), TCRβ (H57-597), CD38 (90), CD206 (C068C2), γδ TCR (GL3), Ki67 (16A8) all Biolegend, and CD4 (RM4.5), CD62L (MEL-14) from BD Biosciences, and MSR1 (M204PA), arginase (A1exF5) from Invitrogen. All antibodies were used at a final concentration of 1µg/mL, except for anti-CD206 and anti-F4/80 (2µg/mL) and anti-MSR1 (4µg/mL).

Validation

All antibodies against surface-expressed markers used in this study have been previously validated by the manufacturer, as stated on their associated product webpages (e.g. https://www.biolegend.com/en-gb/products/pe-anti-mouse-cd45-antibody-100), and by our own lab in previous experiments (e.g. https://doi.org/10.1038/s41467-023-43061-0).

# Animals and other research organisms

Policy information about studies involving animals; ARRIVE guidelines recommended for reporting animal research, and Sex and Gender in Research

Laboratory animals

Wild-type refers to C57BL/6NCrl (Charles River) or the corresponding littermates of genetically-modified lines; Sall1-CreERRosa26Ai14 and Rag2-/-. Rosa26Ai14 mice were originally purchased from Jackson and colonies bred and maintained at the University of Birmingham. Sall1-CreER mice were a kind gift from Dr Melanie Greter (University of Zurich). For adoptive transfer experiments, CnT.II mice17 (a gift from Prof. Kazuyoshi Kawakami, University of Tohoku) were used as donors. Alternatively, CnT.II mice were crossed with Nr4a3-Tocky19 animals that had been crossed with Great-SMART49 cytokine reporter animals. An F1 cross between CnT.II and Nr4a3-Tocky-Great-SMART reporters were used for donors in adoptive transfers. Nr4a3-Tocky mice were developed in Imperial College, London (Ono lab)19. Colonies of CnT.II and Nr4a3-Tocky-Great-SMART were maintained at the Biomedical Service Unit as above.

Wild animals

No wild animals used in the study.

Reporting on sex

Experiments with transgenic mice used male and female littermates. We used female mice for experiments with wild-type mice only, since female mice can be housed in larger groups/smaller cage numbers.

Field-collected samples

N/A

Ethics oversight

All animal studies and associated ethics were approved by the Animal Welfare and Ethical Review Board at the University of Birmingham and under project licences PBE275C33 and PP7564605.

Note that full information on the approval of the study protocol must also be provided in the manuscript.

# Plants

Seed stocks

*Report on the source of all seed stocks or other plant material used. If applicable, state the seed stock centre and catalogue number. If plant specimens were collected from the field, describe the collection location, date and sampling procedures.*

Novel plant genotypes

*Describe the methods by which all novel plant genotypes were produced. This includes those generated by transgenic approaches, gene editing, chemical/radiation-based mutagenesis and hybridization. For transgenic lines, describe the transformation method, the number of independent lines analyzed and the generation upon which experiments were performed. For gene-edited lines, describe the editor used, the endogenous sequence targeted for editing, the targeting guide RNA sequence (if applicable) and how the editor was applied.*

Authentication

*Describe any authentication procedures for each seed stock used or novel genotype generated. Describe any experiments used to assess the effect of a mutation and, where applicable, how potential secondary effects (e.g. second site T-DNA insertions, mosiacism, off-target gene editing) were examined.*

# Flow Cytometry

## Plots

Confirm that:

- [ ] The axis labels state the marker and fluorochrome used (e.g. CD4-FITC).
- [x] The axis scales are clearly visible. Include numbers along axes only for bottom left plot of group (a 'group' is an analysis of identical markers).
- [x] All plots are contour plots with outliers or pseudocolor plots.
- [x] A numerical value for number of cells or percentage (with statistics) is provided.

## Methodology

Sample preparation

Isolated leukocytes were resuspended in PBS and stained with Live/Dead stain (Invitrogen) on ice as per manufacturer's instructions. Fc receptors were blocked with anti-CD16/32 and staining with fluorochrome-labelled antibodies was performed on ice. Labelled samples were acquired immediately or fixed in 2% paraformaldehyde prior to acquisition.

Instrument

Samples were acquired on a 5 laser BD LSR Fortessa.

| | |
|---|---|
| Software | The flow cytometer software used to collect the data was BD FACSDiva, and the final analysis was completed using FlowJo (TreeStar). |
| Cell population abundance | The abundance of cell populations analysed in this study are shown in the Figures and the gating strategies in Fig S2 and S11. Post-sort analyses confirmed the purity of our sorted populations as >95%. |
| Gating strategy | Gating strategies are shown in Fig S2 and S11. |

☒ Tick this box to confirm that a figure exemplifying the gating strategy is provided in the Supplementary Information.
